# Gene-specific machine learning model to predict the pathogenicity of *BRCA2* variants

**DOI:** 10.3389/fgene.2022.982930

**Published:** 2022-09-30

**Authors:** Mohannad N. Khandakji, Borbala Mifsud

**Affiliations:** ^1^ College of Health and Life Sciences, Hamad Bin Khalifa University, Ar-Rayyan, Qatar; ^2^ Hamad Medical Corporation, Doha, Qatar; ^3^ William Harvey Research Institute, Queen Mary University of London, London, United Kingdom

**Keywords:** breast cancer, variant pathogenicity, in-silico predictions, variant prioritization, VUS

## Abstract

**Background:** Existing *BRCA2-*specific variant pathogenicity prediction algorithms focus on the prediction of the functional impact of a subtype of variants alone. General variant effect predictors are applicable to all subtypes, but are trained on putative benign and pathogenic variants and do not account for gene-specific information, such as hotspots of pathogenic variants. Local, gene-specific information have been shown to aid variant pathogenicity prediction; therefore, our aim was to develop a *BRCA2*-specific machine learning model to predict pathogenicity of all types of *BRCA2* variants.

**Methods:** We developed an XGBoost-based machine learning model to predict pathogenicity of *BRCA2* variants. The model utilizes general variant information such as position, frequency, and consequence for the canonical *BRCA2* transcript, as well as deleteriousness prediction scores from several tools. We trained the model on 80% of the expert reviewed variants by the Evidence-Based Network for the Interpretation of Germline Mutant Alleles (ENIGMA) consortium and tested its performance on the remaining 20%, as well as on an independent set of variants of uncertain significance with experimentally determined functional scores.

**Results:** The novel gene-specific model predicted the pathogenicity of ENIGMA *BRCA2* variants with an accuracy of 99.9%. The model also performed excellently on predicting the functional consequence of the independent set of variants (accuracy was up to 91.3%).

**Conclusion:** This new, gene-specific model is an accurate method for interpreting the pathogenicity of variants in the *BRCA2* gene. It is a valuable addition for variant classification and can prioritize unreviewed variants for functional analysis or expert review.

## Introduction

Breast cancer is the most common cancer in women, impacting more than two million each year (Bray et al., 2020; Sung et al., 2021). The disease affects one in seven women worldwide and causes the greatest number of cancer-related deaths among them (McGuire, Brown, Malone, McLaughlin, & Kerin, 2015; Sung et al., 2021). In 2020, it resulted in 684,996 deaths: equal to 15.5% of all cancer deaths among women (Ferlay et al., 2020; Sung et al., 2021). Early breast cancer detection with suitable treatment could reduce breast cancer death rates significantly in the long-term. If the cancer is located only in the breast, the 5-year survival rate of women with breast cancer is 99%, however, if the cancer has spread to a distant part of the body, the 5-year survival rate decreases to 27% (Noone et al., 2018). Therefore, to improve breast cancer outcomes and survival, early detection is crucial. Early detection involves two strategies: screening and early diagnosis. Nevertheless, the balance of potential benefits over risks for mammographic breast cancer screening of the general population is controversial (Canelo-Aybar et al., 2021). A Cochrane review published in 2013 found that it is unclear if mammographic screening does more good or harm (Gøtzsche & KJ, 2013). Recent studies suggest that mammographic screening could be most effective if offered based on the personal risk of the patient calculated from family history, breast density, reproductive factors, demographic, clinical, imaging-related and genetic data (Clift et al., 2021). This highlights the great importance of genetic testing in identifying high risk individuals for screening and early detection. Mutations in several genes were associated with increased risks of breast cancer, according to the Breast Cancer Association Consortium, this includes: *BRCA1, BRCA2, PALB2, CHEK2, ATM, BARD1, MSH6, RAD51C, RAD51D, NF1, TP53* and *PTEN* (Dorling et al., 2021).

BRCA1 and BRCA2 are tumor suppressors that aid in repairing damaged DNA or destroy cells if DNA cannot be repaired (Yoshida & Miki, 2004). These genes are the two major breast and ovarian cancer predisposition genes. Mutations in *BRCA1* and *BRCA2* account for up to 90% of familial breast and ovarian cancer cases (Ford et al., 1998; Mahdavi et al., 2019). The prevalence of mutation in one of those genes was previously estimated to be approximately 1 in every 400 women, nonetheless, recent studies found an overall prevalence of up to 1 in 139 individuals of the general population (Group, 2000; McClain, Palomaki, Nathanson, & Haddow, 2005; Manickam et al., 2018; Abul-Husn et al., 2020). It was estimated that the cumulative breast cancer risk for a 70-year-old woman is up to 87% for *BRCA1* and 84% for *BRCA2* mutation carriers with corresponding ovarian cancer risks up to 68% and 30%, respectively. The prevalence of breast cancer in those females was estimated to be 10–30 times more than in those with no inherited gene mutation (Antoniou et al., 2003; Begg et al., 2008; Brohet et al., 2014; S. Chen et al., 2006; Evans et al., 2008; Ford et al., 1998; Gabai-Kapara et al., 2014; Hopper et al., 1999; Kuchenbaecker et al., 2017; Milne et al., 2008; Park et al., 2021).

Researchers have identified thousands of mutations in *BRCA* genes, some of which were determined to be harmful, while others have no proven impact. The risk associated with any given variant varies significantly and depends on the exact type and location of the variant (Dorling et al., 2022; H. Li et al., 2022; López-Urrutia et al., 2019; Morris & Gordon, 2010). High risk variants typically disrupt the gene function; however, the functional impact of many variants cannot be deduced from their sequence information alone. Such variants are defined as variants of uncertain significance (VUS) and they represent a major challenge for the management of families, in which they are identified (Eccles et al., 2015; López-Urrutia et al., 2019). Worldwide genetic testing has uncovered thousands of VUS in the *BRCA* genes, including missense substitutions, in-frame insertions and deletions, silent alterations that may influence splicing or translation and intronic changes of unknown influence on gene splicing or expression (Spurdle et al., 2012; López-Urrutia et al., 2019; NCBI ClinVar database, 2021).

A consistent variant classification system is essential to the use of genomics in patient care. The 2015 joint recommendation of the American College of Medical Genetics and Genomics and the Association for Molecular Pathology (ACMG/AMP 2015 guidelines) classifies sequence variants into five categories: pathogenic, likely pathogenic, uncertain significance, likely benign, and benign (Richards et al., 2015). For best classification of cancer gene variants, the probability of pathogenicity is based on *in silico* analysis of the sequence alteration in combination with the available genetic, epidemiological and clinical data, such as: segregation analysis, personal and family history, tumor histopathology, and co-occurrence (Lindor et al., 2012; Richards et al., 2015). While several assumptions are made in these calculations, this approach has been widely used to classify variants as pathogenic or benign. It is the currently accepted method for classifying *BRCA* variants by the Evidence-Based Network for the Interpretation of Germline Mutant Alleles Consortium (ENIGMA) that specializes in clinical classification of *BRCA* variants (Spurdle et al., 2012), the ClinVar database of variants, and the Global Alliance for Genomic Health organization in their BRCA Exchange database (Cline et al., 2018). It is noteworthy that there are still >29,000 *BRCA2* variants in the BRCA Exchange database that have not been reviewed for classification.

The number of identified germline variants in *BRCA2* outpace the clinical annotation due to the limited availability of genetic, epidemiological, and clinical data, which highlights the importance and the practicality of computational methods for risk assessment, as well as the need to prioritize *BRCA2* variants for functional testing or classification. In fact, there was a recent call to action to complement the use of the ClinVar database with computational predictors to enhance the actionability of rare breast cancer-gene variants (Saad et al., 2022). Moreover, the existing *BRCA2* pathogenicity prediction algorithms focus on the prediction of the functional impact, as measured by functional assays, of missense variants only. Therefore, our aim is to develop a gene-specific machine learning model to predict pathogenicity according to the comprehensive ACMG guidelines and for all types of *BRCA2* variants, utilizing novel features. We will use this new model to predict the pathogenicity of all *BRCA2* variants that have not been classified yet and prioritize them according to their predicted level of pathogenicity.

## Materials and methods

### *BRCA2* set of variants

We downloaded *BRCA2* variants from the BRCA Exchange database, which contains information drawn from multiple databases that provide a comprehensive list of *BRCA1* and *BRCA2* variants with their annotations (https://brcaexchange.org/variants; accessed on 14 March 2022). It contains variants curated and classified by an international consortium of investigators (ENIGMA consortium) to assess variant pathogenicity. At the time of this study, there were 33,550 *BRCA2* variants, of which 4,102 variants were reviewed by the ENIGMA expert panel and had known effect of being pathogenic (2,672), likely benign (738) or benign (692).

### Variant annotation

The Ensembl Variant Effect Predictor determines the effect of any variant on genes, transcripts, and protein sequence, as well as on regulatory regions. It is a tool for the analysis and annotation of genomic variants. It provides information on the affected transcript, protein, non-coding region, on the frequency and the phenotypes associated with the variant. Additionally, it provides access to numerous *in silico* pathogenicity prediction scores that are present in the dbNSFP database (Liu, Jian, & Boerwinkle, 2011). The *in silico* predictions we included in the model were BayesDel_addAF, BayesDel_noAF, bStatistic, CADD, ClinPred, DANN, Eigen, EigenPC, FATHMM-XF coding, FATHMM-MKL coding, GenoCanyon, GERP++RS, GM12878fitCons, H1hESCfitCons, HUVECfitCons, integratedfitCons, LRT, MaxEntScan, MCAP, MetaLR, MetaSVM, MutationAssessor, MutationTaster2, MutPred, MPC, MVP, phastCons, PhyloP, Polyphen, PrimateAI, PROVEAN, REVEL, SIFT, SiPhy, SpliceAI, and VEST4. For the *in silico* predictions, we used the rank scores whenever they were provided. The detailed list is presented in Table1.

**TABLE 1:** The receiver operating characteristic (ROC) curve analysis for the different in silico predictions. AUC: Area under the curve, Obs: Number of observations, Std.Err: Standard error, CI: Confidence interval.

In silico prediction method	Observation	AUC	Std.Err	95% CI Low	95% CI High
XGBoost	820	1	0	0.99996	1
Consequence	4102	0.9978	0.0009	0.9961	0.99945
IMPACT	4102	0.9986	0.0005	0.99765	0.99957
SIFT_Score	142	0.1166	0.0237	0.07008	0.16318
PolyPhen_Scor	142	0.8811	0.0493	0.78446	0.97781
BayesDel_addAF_rankscore	717	0.9896	0.005	0.97974	0.99946
BayesDel_noAF_rankscore	717	0.9657	0.0105	0.94517	0.98617
CADD_raw_rankscore	717	0.9916	0.0041	0.9836	0.99968
ClinPred_rankscore	142	0.9922	0.0054	0.98165	1
DANN_rankscore	717	0.6899	0.0341	0.6231	0.75676
Eigen-PC-raw_coding_rankscore	717	0.8116	0.0255	0.76169	0.86157
Eigen-raw_coding_rankscore	717	0.8641	0.0225	0.81998	0.90832
FATHMM_converted_rankscore	142	0.9238	0.0437	0.8381	1
GERP++_RS_rankscore	717	0.658	0.0271	0.60495	0.71103
GM12878_fitCons_rankscore	717	0.4681	0.0266	0.41598	0.52016
GenoCanyon_rankscore	717	0.5597	0.0264	0.50789	0.61149
H1-hESC_fitCons_rankscore	717	0.5535	0.0285	0.49759	0.6095
HUVEC_fitCons_rankscore	717	0.5132	0.0257	0.46293	0.56354
LRT_converted_rankscore	717	0.5833	0.0273	0.52976	0.63693
M-CAP_rankscore	120	0.9181	0.0341	0.85119	0.985
MPC_rankscore	141	0.9294	0.0295	0.87164	0.98714
MVP_rankscore	135	0.9563	0.0184	0.92017	0.99247
MetaLR_rankscore	142	0.9414	0.0373	0.86837	1
MetaRNN_rankscore	142	0.995	0.0038	0.98745	1
MetaSVM_rankscore	142	0.9096	0.0688	0.77467	1
MutPred_rankscore	46	0.9821	0.0147	0.95336	1
MutationTaster_rankscore	717	0.984	0.0077	0.96888	0.99921
PROVEAN_converted_rankscore	142	0.596	0.068	0.46263	0.72934
PrimateAI_rankscore	141	0.9153	0.0666	0.78482	1
REVEL_rankscore	142	0.9531	0.0217	0.91052	0.99573
SiPhy_29way_logOdds_rankscore	717	0.6476	0.0266	0.59544	0.69976
VEST4_rankscore	717	0.9948	0.0023	0.99039	0.99925
bStatistic_converted_rankscore	717	0.525	0.0271	0.47193	0.57798
fathmm-MKL_coding_rankscore	717	0.6825	0.0281	0.62749	0.73749
fathmm-XF_coding_rankscore	717	0.4945	0.031	0.43373	0.55523
integrated_fitCons_rankscore	717	0.5015	0.0262	0.45006	0.55286
phastCons17way_primate_rankscore	717	0.5545	0.0287	0.49815	0.61085
phyloP17way_primate_rankscore	717	0.4938	0.0341	0.42692	0.56064
MaxEntScan_alt	64	0.1129	0.0416	0.03141	0.19444
MaxEntScan_diff	64	0.8333	0.0496	0.73613	0.93054
MaxEntScan_ref	64	0.3891	0.0843	0.22388	0.55435
SpliceAI_pred_DS_AG	3655	0.5148	0.0048	0.50537	0.52418
SpliceAI_pred_DS_AL	3655	0.5149	0.0029	0.50917	0.52065
SpliceAI_pred_DS_DG	3655	0.5016	0.0032	0.49532	0.5078
SpliceAI_pred_DS_DL	3655	0.5202	0.0032	0.51396	0.52638

Other variables that were collected or derived from VEP included the position of the variant, variant length (number of bases involved based on reference and alternative alleles), presence in protein domain, variant association with phenotype, presence as a somatic mutation, variant impact, and variant consequences. The variant consequence variable included 18 different effects of the variant position (Supplementary Table S1). We ranked them based on the assumed pathogenicity of the effect with downstream variants having the least effect and stop gained variants having the highest effect.

### Allele frequencies

We obtained population frequency of the variants from both the BRCA Exchange database and from VEP, which included population frequency data from: Exome Aggregation Consortium, NHLBI exome sequencing, 1000 Genomes Project, gnomAD, UK10K cohort data, and the NHLBI Exome Sequencing Project ESP6500 data. We used the highest frequency reported for any given variant as a variable called maximum allele frequency in the model.

### XGBoost

XGBoost (Extreme Gradient Boosting) is an open-source software, which provides a regularizing gradient boosting framework (T. Chen & Guestrin, 2016). It implements a highly flexible, optimized distributed gradient boosting machine learning algorithm under the Gradient Boosting framework through parallel processing to speed up calculations, regularization to avoid overfitting, tree-pruning and handling of missing values.

We chose XGBoost, because it is widely used in bioinformatics; some of those applications were for analyzing protein translocation between cellular organelles (Mendik et al., 2019); predicting gene expression values (W. Li, Yin, Quan, & Zhang, 2019); predicting early-stage prostate cancer (Danciu et al., 2020); identifying the origin of DNA replication (Do & Le, 2020); and predicting Kruppel-like factors (Le, Do, & Le, 2021). Additionally, XGBoosted Machine learning performed better than other predictive models, including Linear models, Gradient Boosting Machines, Neural Networks, Random Forests, and Extremely Randomized Forests, in predicting the functional impact of *BRCA2* missense variants (Hart, Polley, Shimelis, Yadav, & Couch, 2020).

### Model building

We used the XGBoost R package (version 1.4.1.1) with default parameters (booster = “gbtree”, objective = “binary:logistic”, eta = 0.3, gamma = 0, max_depth = 6, min_child_weight = 1, subsample = 1, colsample_bytree = 1, nrounds = 100) to train a classifier model on the variant annotations for predicting pathogenicity. Pathogenicity was based on the ENIGMA expert panel’s review. Therefore, only the variants that have been reviewed were included in building the model (4,012 variants), and they were split into 80% training set and 20% test set. The original variant pathogenicity groups were recategorized as pathogenic (“pathogenic” and “likely pathogenic”) and benign (“benign” and “likely benign”) for the binary classification. The model was trained to predict the expert classification of either pathogenic or benign variants and we performed 5-fold cross-validation of the model. We used the xgb.plot.importance function to show which are the top 10 most important features of the model (gain was used as the measure of importance). The Shapely values were also examined to find the most predictive characteristics and prediction scores (xgb.plot.shap function and SHAPforxgboost package). Finally, we predicted the pathogenicity of the 29,448 unreviewed variants.

### Testing the model on independent VUS with functional data

Richardson et al., in 2021 assessed the functional effect of 252 *BRCA2* VUS by a *BRCA2* homology-directed DNA repair (HDR) assay. Utilizing the Variant Recoder tool in Ensembl, the 252 BRCA2 amino acid changes corresponded to 276 missense sequence variants. Out of the 276 variants, 251 were not reviewed by the BRCA Exchange database and 4 of them were both missense and splice region variants. Accordingly, 247 variants were used for independent assessment of the model on missense VUS. Those VUS had functional data on their ability to complement DR-GFP BRCA2 deficient V-C8 cells in a BRCA2 homology-directed DNA repair (HDR) assay. Known pathogenic variants with functional defects had HDR scores <1.66, and known benign variants that were nonfunctional had HDR scores >2.44 (Richardson et al., 2021). More extreme HDR scores of <1.0 and >3.0 have also been utilized in the literature for pathogenic and benign variants, respectively (Guidugli et al., 2018), therefore we tested the model’s performance with both cut-offs.

## Results

### Variant datasets

At the time of data collection, the BRCA Exchange database had 33,550 *BRCA2* variants. The largest proportion of those variants were intronic (36%), and of those found in the coding region, the majority were missense (53%: Supplementary Figures S1A,B). Only 4,102 variants were reviewed by the expert panel and had a known effect: 2,672 were pathogenic, and 1,430 were benign. The distribution of the variants in the expert reviewed portion markedly differed from the distribution of all variants. Out of the reviewed 4,102 variants, 29% were frame shift, compared to the 5% of all variants, and intronic variants were only 9%, compared to the 36% of all variants (Supplementary Figures S1A,C). There was an even more pronounced distribution difference for coding sequence variants, with the proportion of frameshift, synonymous and stop gained variants being much higher for the reviewed variants, while only 4% of them were missense variants (Supplementary Figures S1B,D). This highlights that a large proportion of missense variants could not be unambiguously assigned to either pathogenic or benign categories.

### Variant location

**FIGURE 1:**
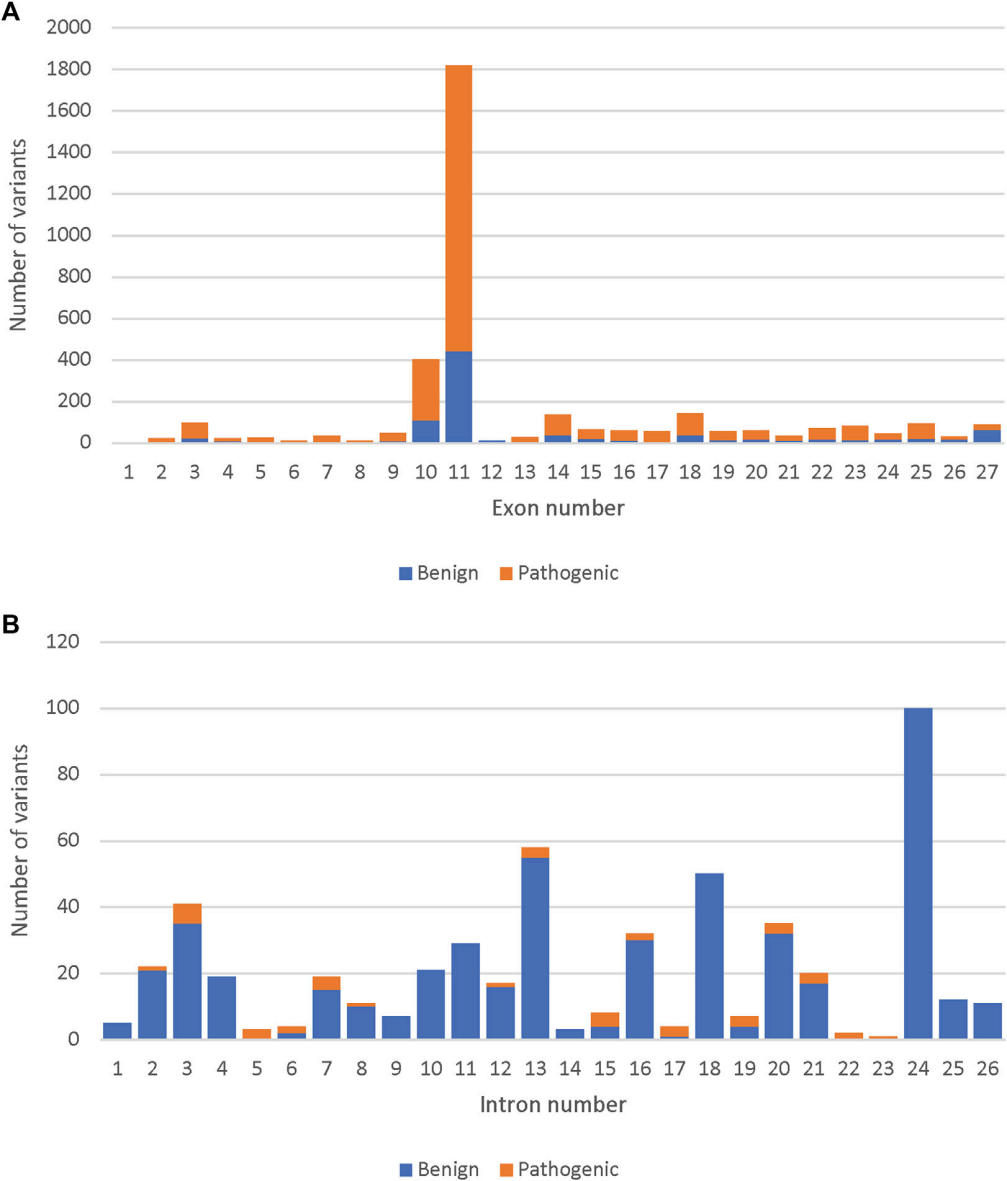
Comparison of the number of pathogenic and benign variants among the 4,102 reviewed, across the BRCA2 gene. **(A)** The number of pathogenic and benign variants per BRCA2 exons. **(B)** The number of pathogenic and benign variants per BRCA2 introns.

Out of the 33,550 *BRCA2* variants, 14,259 were present along the 27 *BRCA2* exons. Out of the 4,012 reviewed variants, the highest number of both benign and pathogenic variants were found in exon 11 (47.6%) followed by exon 10 (10.4%) (Figure 1A). Similarly, the highest number of specifically missense variants were present in Exons 10 and 11 (41.1% and 15.6% of all missense variants, respectively). However, exon 10 and 11 had only benign missense variants. Pathogenic missense variants were present in exons 13, 17, 18, 24 and 25 (Supplementary Figure S2). Only a small fraction of the reviewed variants were intronic, and most of those were determined to be benign (Figure 1B).

### Predicting pathogenicity of the ENIGMA reviewed variants

To develop the prediction model, we used an extreme gradient boosting machine learning algorithm (XGBoost) and included the variants with known expert reviewed effect (4,102 variants). Variants were divided into a training (80%) and a test set (20%). The training model included 3,282 variants and it was trained to predict the expert classification of either pathogenic (2,118 variants) or benign (1,164 variants) variants. The test model included 554 pathogenic and 266 benign variants.

**FIGURE 2:**
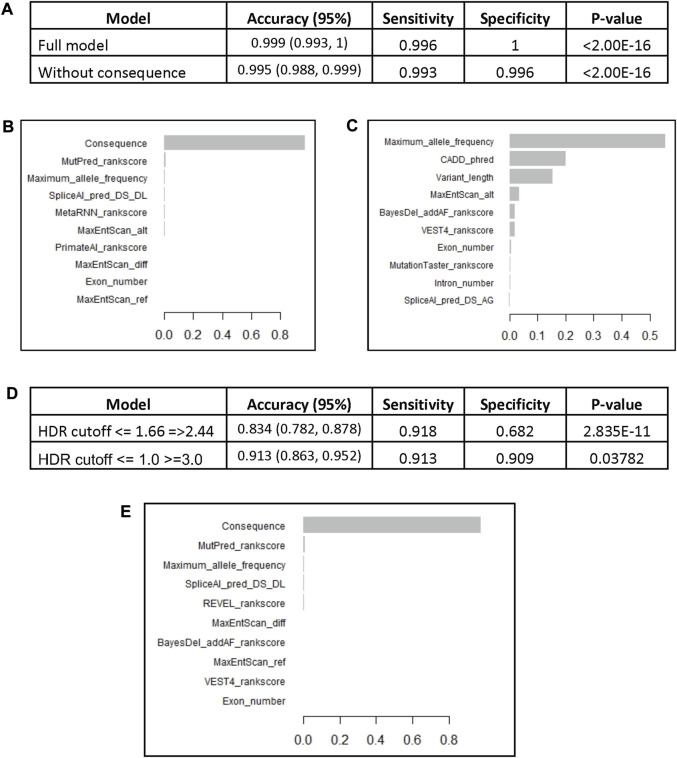
The BRCA2 XGBoost models. **(A)** The models characteristics (AUC: Area Under the Curve). **(B)** Feature importance of the XGBoost model. **(C)** Feature importance of the XGBoost model without consequence. The BRCA2 XGBoost model trained on the whole reviewed dataset (4,102 variants) was used to predict VUS pathogenicity based on HDR functional assay scores. **(D)** The performance of the model on a set of pathogenic and benign variants according to HDR cutoffs <= 1.66 and =>2.44 (247 variants) and cutoffs <= 1.0 and >=3.0 (160 variants). **(E)** Feature importance of the XGBoost model trained on the whole reviewed dataset.

The model was used to predict the test group of 820 variants and yielded an accuracy of 0.999 with sensitivity of 99.6% and the specificity of 100% (Figure 2A). The most important variable was the variant consequence followed by a combination of different *in silico* prediction tools (Figure 2B, Supplementary Figure S3A). Removing the consequence variable from the model did not affect the accuracy (0.996) and the maximum allele frequency became the most important feature followed by the Combined Annotation Dependent Depletion (CADD) Phred score and the number of involved bases “variant length” (Figure 2C, Supplementary Figure S3B). We performed cross validation of the *BRCA2* model with 5 different subsamples that included random training and test groups. All models demonstrated similar accuracies between 99.6% and 99.9% (Supplementary Table S2). Similar to the original model, the variant consequence was the most important variable across the 5 subsamples, and when it was removed, the maximum allele frequency became the most important feature.

### Comparison of the novel model to previous *in silico* prediction algorithms

We compared the area under the curve for our novel XGBoost model and the input *in silico* prediction tools on their own. The XGBoost model had the highest AUC of 1.00, followed by VEST4, ClinPred, and CADD rank scores (Table 1). It should be noted that, the receiver operating characteristic (ROC) analysis of the different *in silico* predictions was performed using the full sample of 4,102 variants and the AUCs were calculated for only those variants that had prediction scores for the given tool.

We also performed ROC analysis for the association of the consequence, which demonstrated excellent diagnostic abilities with the area under the curve (AUC) equal to 99.8% (Supplementary Figure S4). The cutoff point of more than 12 (missense variant) had the best balance between sensitivity (99.4%) and specificity (99.9%). Thus, we can expect that the model’s accuracy in identifying only VUS will decrease, because VUS are usually missense variants.

### Model validation in predicting VUS

We obtained missense VUS with functional data from a recent study by Richardson et al. After removing variants that were already included in building the model, 247 variants were used to assess the model’s performance in predicting variants of uncertain significance. Out of 247 VUS, 88 demonstrated functional defects with HDR scores <1.66, and 159 variants were considered benign with HDR >2.44.

The *BRCA2* model, trained on the full set of ENIGMA *BRCA2* variants (4,102), was tasked to predict the VUS that demonstrated functional defects. The model had high accuracy of 0.834 with sensitivity of 91.8% and specificity of 68.2% (Figure 2D). The most important variable was the variant consequence (Figure 2E, Supplementary Figure S5). The diagnostic performance of the model significantly improved with the more extreme HDR cutoff points of <= 1 for pathogenic and >=3 for benign variants (160 variants). The accuracy of the model increased to 0.913 with a sensitivity of 91.3% and specificity of 90.9% (Figure 2D).

### Model pathogenicity predictions for the not reviewed variants

**FIGURE 3:**
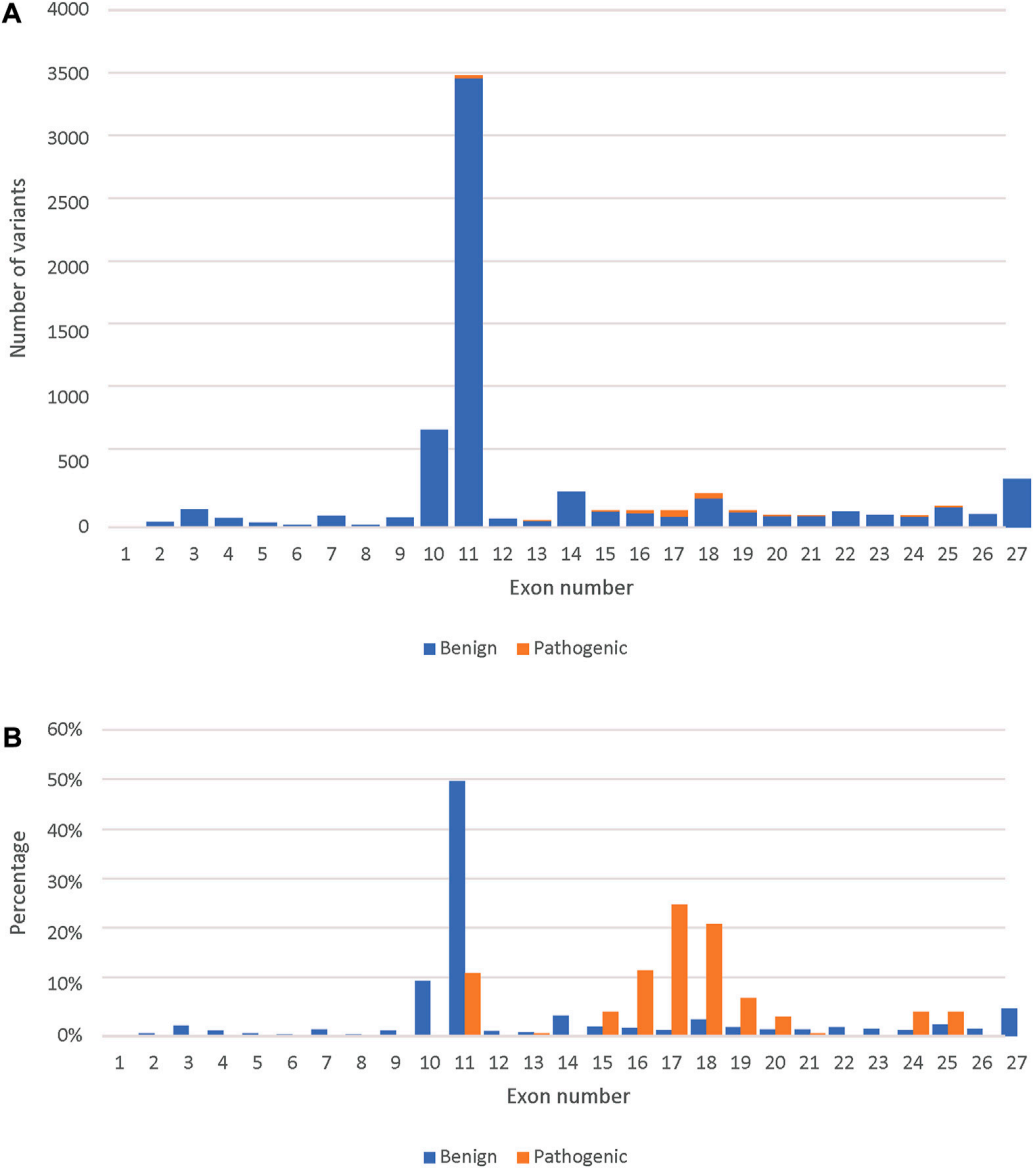
Comparison between the predicted pathogenic and benign variants across the BRCA2 exons. **(A)** The number of predicted pathogenic and benign missense variants (7,131) per BRCA2 exons. **(B)** Percent distribution of predicted pathogenic and benign missense variants across the BRCA2 exons.

Finally, we used the novel gene-specific *BRCA2* model to predict the remaining 29,448 variants present in the BRCA Exchange database that are not yet reviewed by the expert panel. We predicted 2,092 variants to be pathogenic and prioritized them according to the total SHAP values of the different predictors (Supplementary Table S3). We predicted 186 pathogenic missense variants (Figure 3A). The majority of those are in the DNA-binding domain (exons 12–26), however 23 were in exon 11, which is outside of it (Figure 3B).

## Discussion

The highest number of both benign and pathogenic variants were found in exon 11 followed by exon 10, which was expected as those two exons represent around 65% of the *BRCA2* coding sequence. However, looking only at expert reviewed missense variants, exons 10 and 11 had only benign missense variants. This is in agreement with previous studies that referred to exon 10 and 11 as “coldspots” which were described as “regions of a gene that are tolerant of variation, where pathogenic missense variants are unlikely” (Dines et al., 2020). However, we predicted 23 pathogenic missense variants in exon 11, which fell into the BRCA2 BRC repeats that binds to RAD51 and DSS1 resulting in the RAD51–BRCA2–DSS1 complex (Shailani, Kaur, & Munshi, 2018), indicating that these missense variants are likely to effect the complex’s stability. This suggests that only exon 10 is a “coldspot”.

We have demonstrated that the gene-specific *BRCA2* model is an extremely accurate method for predicting variant pathogenicity in the *BRCA2* gene according to the classification by the ENGIMA group. Moreover, the model demonstrated excellent abilities in predicting damaging missense variants of uncertain significance. The gene-specific model demonstrated better diagnostic probabilities than other *in silico* prediction tools. In contrast to other gene-specific models or *in silico* predictions, our model was built to predict the ENGIMA final classification (Hart et al., 2019; Hart et al., 2020). Therefore, it encompasses not only missense variants that are tested in functional studies but all possible variant types. The previously published models or predictions are built specifically for missense variants and to predict their functional impact as tested by functional assays. In fact, the *BRCA2* model developed by Hart *et al.* was limited to missense mutations in the DNA-binding domain of the BRCA2 protein known to be associated with impaired function (Hart et al., 2020). Moreover, the existing *BRCA2* model was trained and tested with only 202 *BRCA2* variants. It is based on small numbers of known damaging mutations, which limits both the model’s ability to capture the variability of variant data and the direct comparison between the two gene-specific models. Nevertheless, to compare our model to the previous one, we examined the performance of our model to predict only the missense variants, which are present in the testing group and calculated the Matthews Correlation Coefficient (MCC). Our model had an MCC of 0.849 which is better than the MCC of 0.73 reported for the previous *BRCA2* model (Hart et al., 2020).

Despite the increasing number of variants that have been functionally tested, there are still 29,448 *BRCA2* variants that have not been classified by the BRCA Exchange expert panel (ENIGMA). Variant classification is based on the probability of pathogenicity that includes *in silico* analysis of the sequence alteration in combination with the available genetic, epidemiological, and clinical data, as well as functional studies (Lindor et al., 2012). All these underscore the importance and current need of computational methods to predict and prioritize variants for classification or functional testing. Our prioritized list of so far unreviewed variants could guide future efforts in studying damaging mutations and aid genetic counselors and researchers for interpreting the pathogenicity of different *BRCA2* variants.

There are still limitations for the *BRCA2* model. The fact that variants at the lower and upper extremes of HDR scores had better predictions emphasize that variants with intermediate HDR scores are more challenging for the model. These variants are also likely to represent a set of variants with variable functional effect that might depend on other variants or external factors. Moreover, we did not optimize the model parameters, therefore the model might perform better with optimal settings. We also did not systematically test whether leaving out certain *in silico* prediction variables would improve the model’s performance. While a model with an optimal set of variables might exist, the XGBoost algorithm is resistant to redundant information, and therefore we do not foresee a significant improvement over including all available *in silico* predictions.

The *BRCA2* gene-specific model is an accurate method for interpreting the pathogenicity of all types of variants in the *BRCA2* gene as they were classified according to the ACMG criteria. It is a valuable addition for variant classification and can prioritize unreviewed variants for functional analysis or expert review. Finally, our approach could be utilized for other high-risk cancer genes that have a large number of variants with high-confidence pathogenicity annotation.

## Data Availability

The original contributions presented in the study are included in the article/Supplementary Material, further inquiries can be directed to the corresponding author.
