# Supplementary material for: Gene-specific machine learning model to predict the pathogenicity of BRCA2 variants
Source: Front Genet. 2022 Sep 30;13:982930. doi: 10.3389/fgene.2022.982930 (PMC9561395; doi:10.3389/fgene.2022.982930)
Supplement: Supplementary file 2 [file DataSheet1.docx]

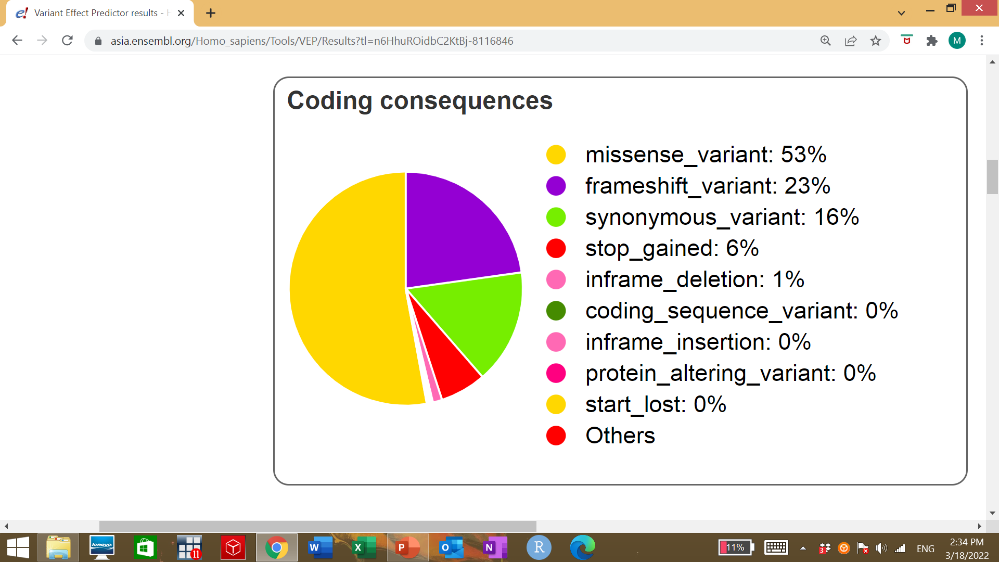

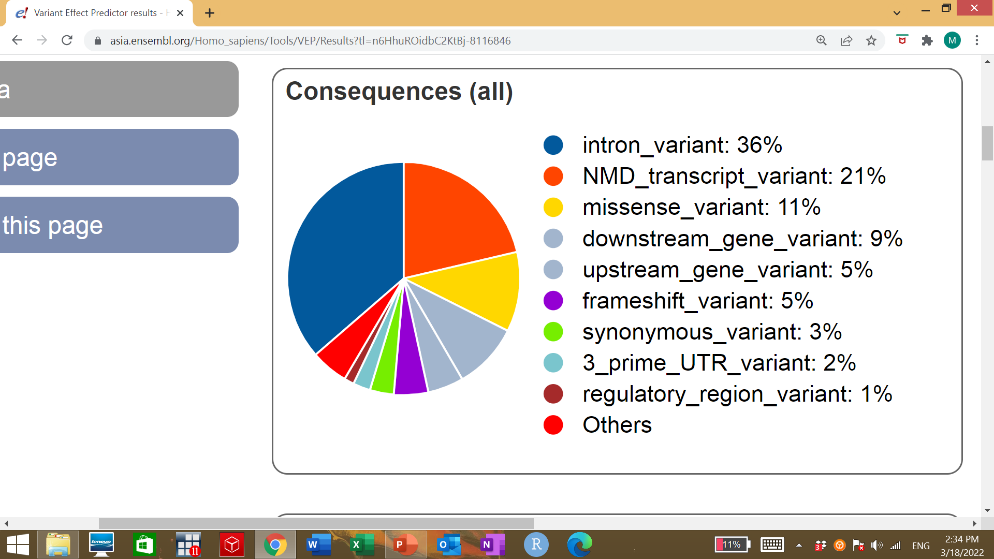

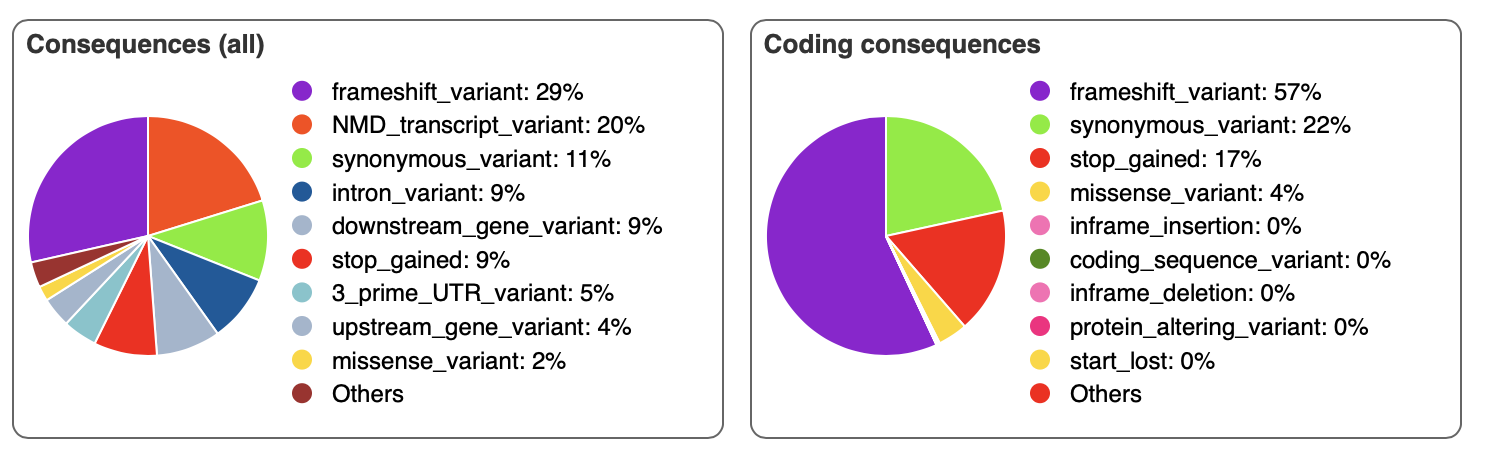


C

D

A

B

**Supplementary Figure 1.** Distribution of consequences according to the Ensembl Variant Effect Predictor for variants downloaded from *BRCA* exchange database. A) Consequences of all *BRCA2* variants. B) Consequences of all coding variants. C) Consequences of *BRCA2* variants that were classified by the expert panel and were used to build the models. D) Consequences of coding variants that were classified by the expert panel.

**Supplementary Figure 2.** Comparison of the percent distribution of the 141 pathogenic and benign reviewed missense variants across the *BRCA2* exons.

SHAP value

SHAP value


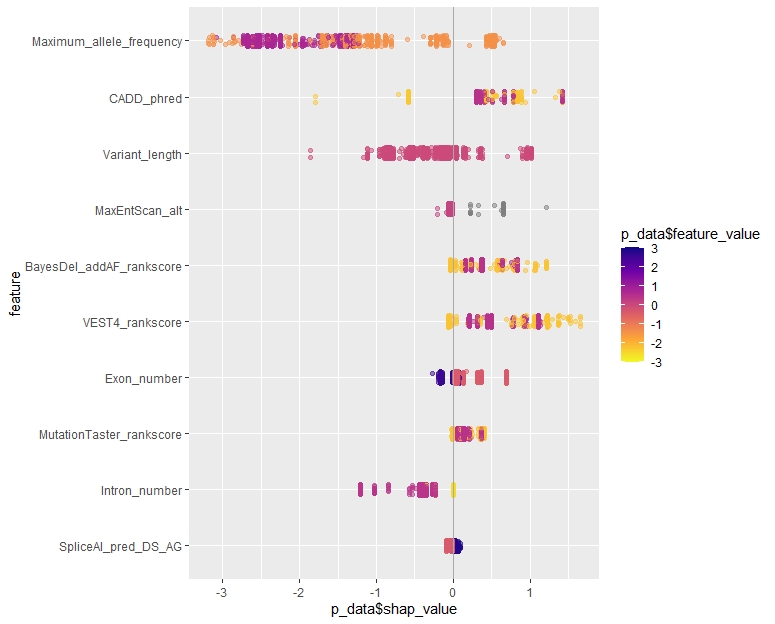

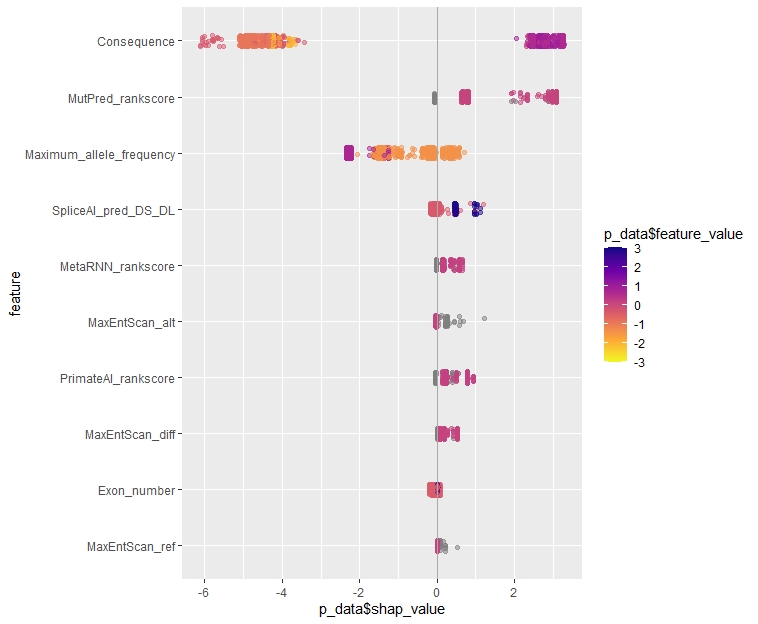


A

B

feature value


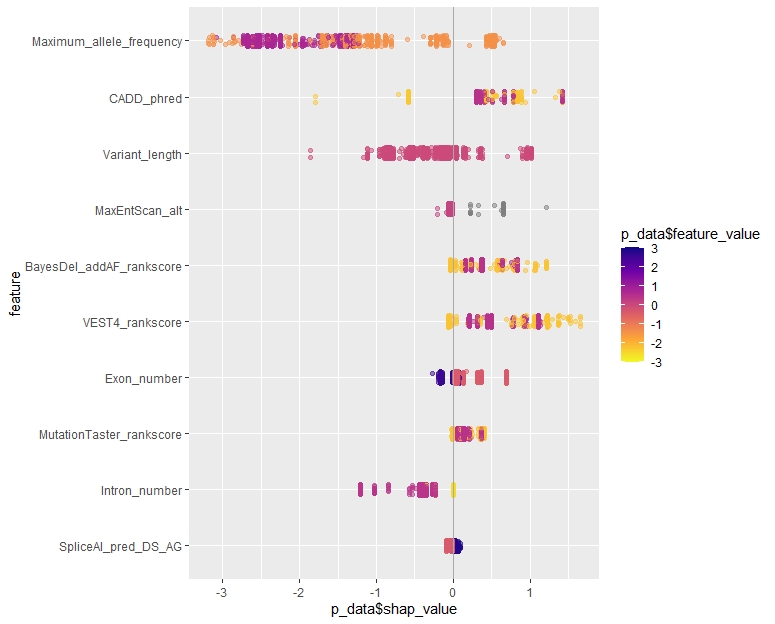


**Supplementary Figure 3.** The *BRCA2* XGBoost models. A) SHAP values for the features in the full XGboost model. B) SHAP values for the features of the XGBoost model without consequence.


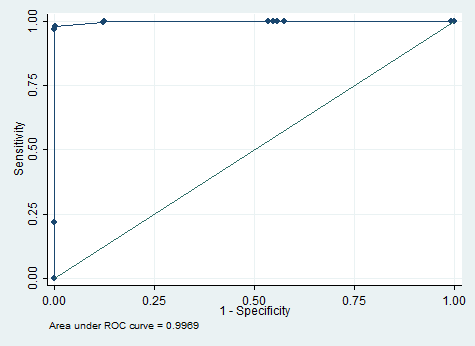


**Supplementary Figure 4.** The receiver operating characteristic curve (ROC curve) of the consequence as a measure of variant pathogenicity.


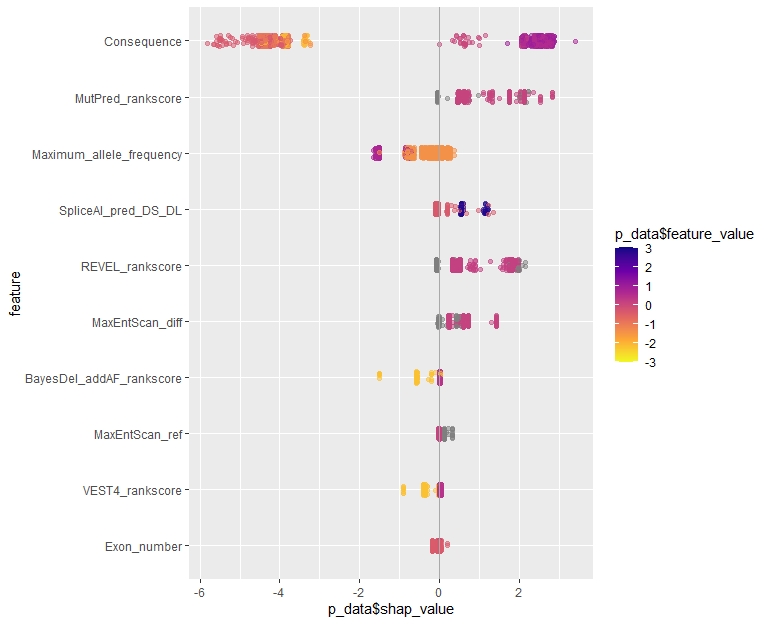


feature value

SHAP value


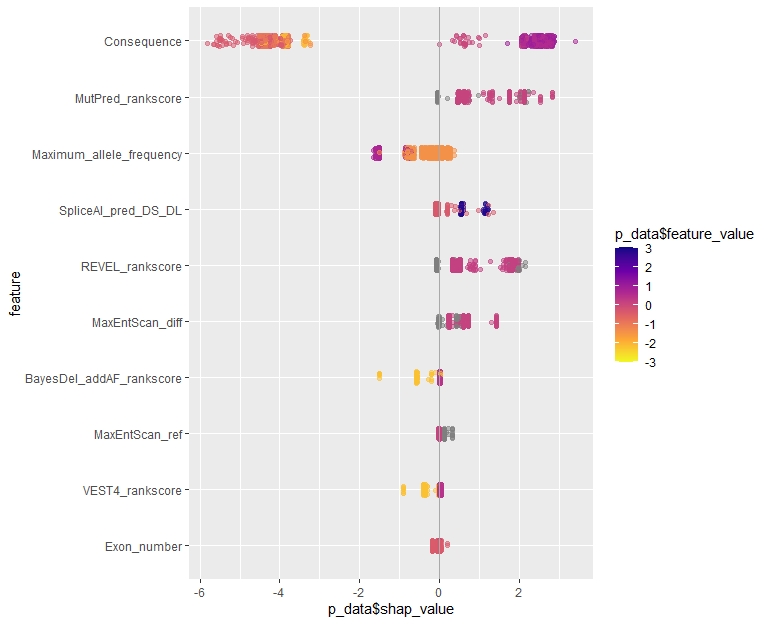


**Supplementary Figure 5.** The SHAP values of the *BRCA2* XGBoost model that was used to predict functional assay results for variants uncertain significance.
